# Prognostic Value of Dynamic Hematologic Immune-Inflammatory Indices in Patients Undergoing Isolated Coronary Artery Bypass Grafting

**DOI:** 10.3390/jcm15187064

**Published:** 2026-09-11

**Authors:** Soner Kumcu, Eren Oral Kalbisağde, Burak Toprak

**Affiliations:** 1 Department of Cardiovascular Surgery, Niğde Ömer Halisdemir Training and Research Hospital, Niğde 51240, Turkey; soner.kumcu@saglik.gov.tr; 2 Department of Cardiovascular Surgery, Faculty of Medicine Hospital, Mersin University, Mersin 33110, Turkey; drkalbisade@mersin.edu.tr; 3 Department of Cardiovascular Surgery, Mersin City Education and Research Hospital, Mersin 33240, Turkey

**Keywords:** coronary artery bypass grafting, delta neutrophil index, monocyte-to-lymphocyte ratio, pan-immune-inflammation value, in-hospital mortality, postoperative complications, immune-inflammatory biomarkers

## Abstract

**Background**: Despite advances in surgical and perioperative care, early mortality and postoperative complications after isolated coronary artery bypass grafting (CABG) remain clinically relevant. Conventional risk models primarily reflect baseline clinical and operative characteristics and may not fully capture the dynamic immune-inflammatory response to cardiopulmonary bypass and surgical trauma. Hematologic indices derived from routine complete blood counts may provide a practical and low-cost assessment of this perioperative inflammatory burden. **Methods**: This single-center retrospective observational study included 650 adult patients who underwent isolated CABG between 2020 and 2024. Preoperative and postoperative monocyte-to-lymphocyte ratio (MLR), pan-immune-inflammation value (PIV), and delta neutrophil index (DNI) were evaluated. The primary outcome was in-hospital mortality, and the secondary outcome was a composite postoperative adverse event comprising prolonged mechanical ventilation, prolonged intensive care unit stay, prolonged inotropic support, pneumonia, or sepsis. **Results**: In-hospital mortality occurred in 52 patients (8.0%), and composite postoperative adverse events occurred in 221 patients (34.0%). Non-survivors were older and had higher clinical risk scores, lower ejection fraction, longer cardiopulmonary bypass and aortic cross-clamp times, and less favorable renal and inflammatory profiles. Preoperative and postoperative MLR, PIV, and DNI were significantly higher in non-survivors after false discovery rate correction. In univariable analyses, DNI showed the strongest associations with mortality; however, none of the immune-inflammatory indices remained independently associated with mortality or composite postoperative adverse events after multivariable adjustment. Among individual biomarkers, postoperative DNI showed the highest discrimination for in-hospital mortality (AUC 0.765, 95% CI 0.692–0.838), followed by preoperative DNI (AUC 0.738). Addition of postoperative DNI to the clinical mortality model increased the AUC from 0.805 to 0.815, although the incremental improvement was not statistically significant. **Conclusions**: Although perioperative MLR, PIV, and DNI were associated with adverse outcomes in unadjusted analyses, none remained independently associated with in-hospital mortality or composite postoperative adverse events after multivariable adjustment. Postoperative DNI showed the strongest individual discrimination for mortality; however, its addition to the clinical model resulted in only a small and statistically non-significant improvement in AUC. These findings indicate limited incremental prognostic value of the evaluated immune-inflammatory indices beyond established clinical and operative risk factors and do not support their use as stand-alone predictors after isolated CABG.

## 1. Introduction

CABG continues to be an established revascularization strategy for appropriately selected patients with multivessel disease, left main coronary involvement, or complex coronary anatomy [1]. Improvements in operative techniques, myocardial protection, anesthesia, and postoperative intensive care have reduced perioperative risk; nevertheless, early adverse outcomes after isolated CABG remain clinically important [2]. Postoperative complications including extended ventilatory support, prolonged intensive care treatment, persistent inotropic requirement, pneumonia, and sepsis are associated with greater healthcare utilization and unfavorable early outcomes [1].

The pathophysiology of complications developing after coronary artery bypass surgery often cannot be explained by a single mechanism. Surgical trauma, cardiopulmonary bypass, blood contact with artificial surfaces, ischemia–reperfusion injury, endothelial activation, the complement system, the coagulation cascade, neutrophil activation, and cytokine release are the main processes that trigger the systemic immune-inflammatory response in the early postoperative period [3]. Although this response is part of physiological repair, when it becomes excessive or uncontrolled, it may result in capillary leakage, vasoplegia, myocardial dysfunction, pulmonary complications, renal injury, infectious complications, and multiple organ dysfunction [4]. Therefore, assessing risk after coronary bypass surgery not only on the basis of preoperative clinical characteristics but also together with the magnitude of the inflammatory response emerging during the perioperative period may provide a more comprehensive approach [3].

Currently, clinical risk models such as the STS score, EuroSCORE, and similar tools are widely used to predict perioperative risk in cardiac surgery patients [5]. However, these models are largely based on age, comorbidity burden, ventricular function, type of operation, and certain basic clinical variables. This static structure may not always adequately reflect the dynamic immune-inflammatory response that develops in the postoperative period. Patients with comparable baseline risk may nevertheless experience substantially different postoperative trajectories regarding inflammation, infection, hemodynamic support, ventilation requirements, and intensive care duration [6]. Such variability has encouraged investigation of inexpensive and readily obtainable biomarkers as potential supplements to conventional risk assessment.

Hematologic immune-inflammatory indices derived from the complete blood count have attracted increasing interest in recent years as practical indicators of systemic inflammation in cardiovascular diseases and surgical populations [7]. The monocyte-to-lymphocyte ratio is a simple parameter that reflects the balance between monocyte-mediated inflammatory activation and lymphocyte-mediated immune regulation [8]. The pan-immune-inflammation value aims to summarize the innate immune response, thromboinflammatory burden, and lymphopenia-related immune suppression within a single index by jointly evaluating neutrophil, platelet, monocyte, and lymphocyte components [9]. The delta neutrophil index, by reflecting the circulating immature granulocyte fraction, may provide information on neutrophilic activation, bone marrow response, and early infectious/inflammatory stress [10]. The common advantage of these indices is that they can be calculated from routine hemogram parameters and integrated into perioperative risk assessment without requiring additional cost.

Nevertheless, the available literature shows that inflammatory indices after coronary artery bypass surgery have often been evaluated using single-time-point measurements or a limited number of outcomes [7,8,9,10]. Evidence remains limited regarding studies that simultaneously examine perioperative changes in MLR, PIV, and DNI within a single cohort and directly compare their ability to identify both in-hospital death and clinically relevant postoperative complications [11,12]. This gap makes it important to determine which inflammatory parameter is truly stronger, more consistent, and capable of providing incremental value to the clinical model for early risk stratification after coronary bypass surgery.

In this study, we aimed to evaluate the association of preoperative and postoperative MLR, PIV, and DNI values with in-hospital mortality and composite postoperative adverse events in patients undergoing isolated coronary artery bypass grafting. The primary outcome of the study was defined as in-hospital mortality, whereas the secondary outcome was defined as a composite postoperative adverse event consisting of prolonged mechanical ventilation, prolonged intensive care unit stay, prolonged inotropic support, pneumonia, and sepsis. In addition, by examining the contribution of adding these indices to clinical risk models in terms of discrimination, calibration, and clinical risk classification, we aimed to demonstrate the potential role of dynamic hematologic immune-inflammatory assessment in early postoperative risk prediction.

## 2. Materials and Methods

### 2.1. Data Collection

**Study Design and Population:**

This single-center, retrospective, observational cohort study included consecutive adult patients who underwent isolated coronary artery bypass grafting (CABG) at the Department of Cardiovascular Surgery, Mersin University Faculty of Medicine Hospital between 2020 and 2024. The study aimed to evaluate the prognostic value of preoperative and postoperative hematologic immune-inflammatory indices for in-hospital mortality and early postoperative adverse events.

Patients aged ≥18 years undergoing isolated on-pump CABG with available preoperative and 24 h postoperative complete blood count data, including the components required to calculate monocyte-to-lymphocyte ratio (MLR), pan-immune-inflammation value (PIV), and delta neutrophil index (DNI), and complete outcome data were eligible. During the study period, 812 patients underwent CABG and were screened for eligibility. A total of 162 patients were excluded: 42 underwent emergency or urgent CABG, 38 underwent concomitant cardiac procedures such as valve or aortic surgery, 16 underwent redo CABG, 18 underwent off-pump CABG, 15 had active infection or a clinically significant inflammatory disease, 9 had a hematologic disorder or active malignancy, and 24 lacked the required preoperative or postoperative laboratory data. After application of these prespecified exclusion criteria, 650 patients constituted the final analytic cohort. Accordingly, the final study cohort consisted exclusively of primary, elective, isolated on-pump CABG procedures; emergency or urgent operations, redo procedures, off-pump CABG, and concomitant cardiac operations were not included.

**Figure 1:**
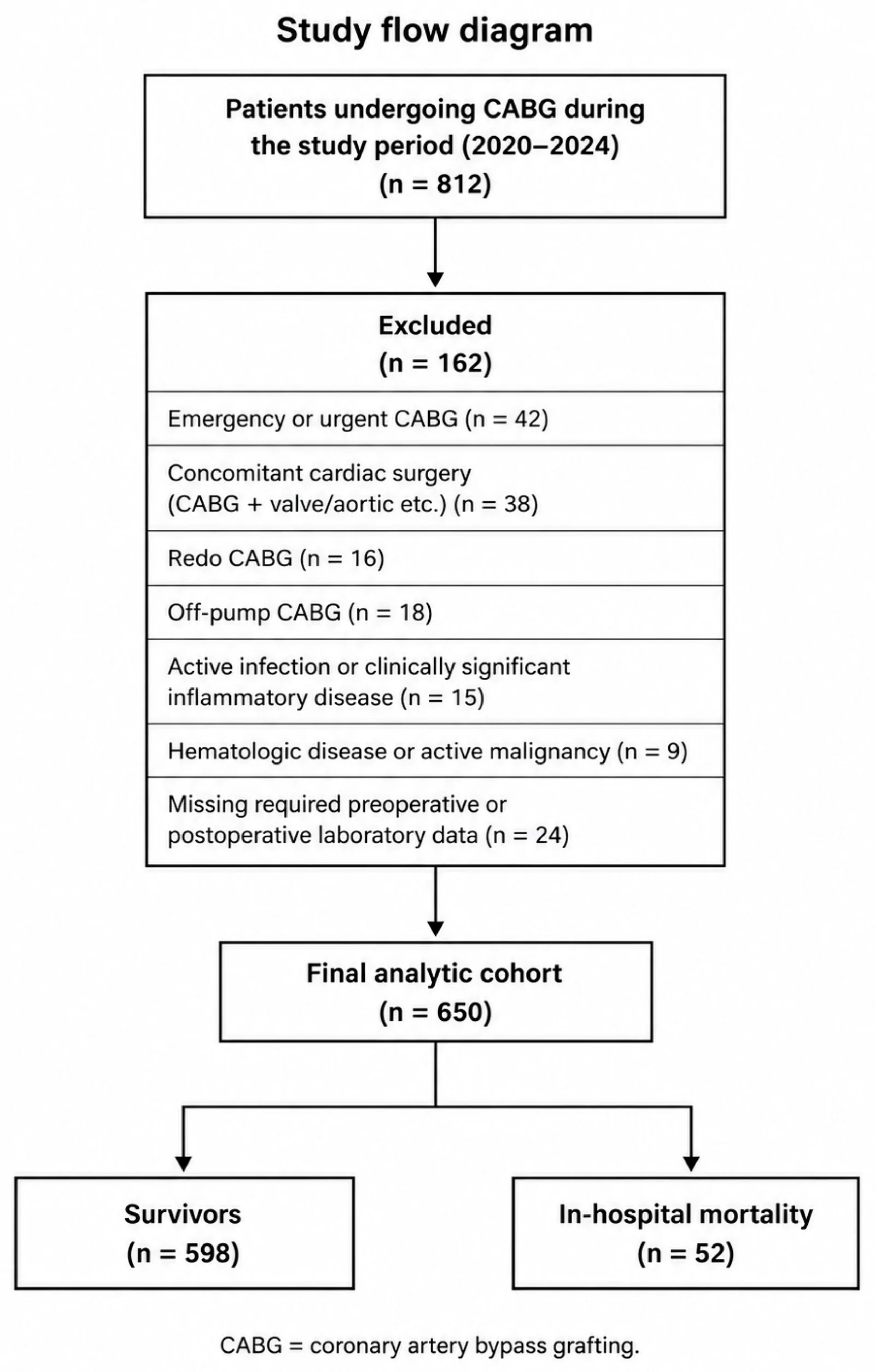
Flow diagram of patient screening, exclusion, and inclusion in the final analytic cohort. CABG = coronary artery bypass grafting.

The final cohort comprised 598 survivors and 52 patients who experienced in-hospital mortality. Survivors served as the comparison group within the same surgical cohort rather than as healthy controls (Figure 1).

The study protocol was approved by the Mersin University Faculty of Medicine Clinical Research Ethics Committee (approval date: 18 September 2024; decision number: 2024/880) and was conducted in accordance with the principles of the Declaration of Helsinki.

**Data Collection and Variable Definitions:**

Data were retrospectively obtained from electronic medical records, patient files, operative and anesthesia records, intensive care unit records, and the laboratory information system. Demographic characteristics, comorbidities, cardiac risk scores, laboratory parameters, operative variables, postoperative course, and in-hospital outcomes were recorded.

Preoperative laboratory values were obtained from routine samples collected before surgery, whereas postoperative values were derived from routine blood samples obtained 24 h after surgery. For analyses involving postoperative biomarkers, the 24 h postoperative blood sampling was considered a landmark time point. Because availability of the postoperative complete blood count was required for inclusion, the analytic cohort was restricted to patients who were alive at the 24 h sampling time point and had the hematologic components required to calculate postoperative MLR, PIV, and DNI. Patients who died before this landmark and consequently lacked the scheduled 24 h postoperative laboratory measurements were not eligible for inclusion in the analytic cohort. Accordingly, associations between postoperative biomarkers and in-hospital mortality should be interpreted as conditional on survival to 24 h after surgery.

Clinical variables included age, sex, diabetes mellitus, hypertension, left ventricular ejection fraction (EF), STS score, EuroSCORE, SYNTAX score, cardiopulmonary bypass (CPB) time, and aortic cross-clamp time.

Routine hematologic measurements included hemoglobin, hematocrit, white blood cell, platelet, neutrophil, lymphocyte and monocyte counts, platelet distribution width, red cell distribution width, mean platelet volume, and DNI. Biochemical variables included urea, creatinine, glomerular filtration rate, sodium, potassium, alanine aminotransferase, aspartate aminotransferase, C-reactive protein, albumin, troponin, and creatine kinase-MB.

MLR was calculated as the absolute monocyte count divided by the absolute lymphocyte count. PIV was calculated as neutrophil count × platelet count × monocyte count/lymphocyte count [9]. DNI was obtained directly from the automated hematology analyzer as an indicator of circulating immature granulocyte/neutrophilic activation. MLR, PIV, and DNI were evaluated separately in the preoperative and postoperative periods.

The primary outcome was in-hospital mortality, defined as all-cause death occurring after surgery during the index hospitalization and before discharge. The secondary outcome was a composite postoperative adverse event, defined as the occurrence of at least one of the following events after the 24 h postoperative biomarker assessment: prolonged mechanical ventilation, prolonged intensive care unit (ICU) stay, prolonged inotropic support, pneumonia, or sepsis. Prolonged mechanical ventilation was defined as the requirement for invasive mechanical ventilation for >24 h after surgery, including additional ventilation time after reintubation. Prolonged ICU stay was defined as an ICU length of stay >48 h after surgery. Prolonged inotropic support was defined as the requirement for continuous intravenous inotropic or vasoactive-inotropic therapy for >24 h after surgery. Postoperative pneumonia was diagnosed on the basis of compatible clinical and radiological findings, including a new or progressive pulmonary infiltrate or consolidation accompanied by clinical evidence of lower respiratory tract infection, such as fever, leukocytosis or leukopenia, purulent respiratory secretions, or worsening oxygenation, in accordance with CDC/NHSN criteria. Sepsis was defined according to the Sepsis-3 criteria as suspected or documented infection associated with acute organ dysfunction, operationalized as an increase in the Sequential Organ Failure Assessment (SOFA) score of ≥2 points attributable to infection. Individual components and cumulative complication burden were also evaluated.

The 24 h postoperative blood sample preceded assessment of the secondary outcomes. Postoperative adverse events were subsequently evaluated during the remaining index hospitalization, and only events occurring or meeting the predefined criteria after the 24 h blood sampling were included in the composite endpoint. Therefore, postoperative MLR, PIV, and DNI measurements temporally preceded the secondary outcome assessment.

Surgical and perioperative management followed the institution’s standard CABG protocols. CPB and aortic cross-clamp durations were obtained from operative records and analyzed as objective measures of operative burden.

### 2.2. Data Analysis

**Statistical Analysis:**

Before analysis, data were checked for coding errors, duplicate records, unit consistency, biological plausibility, outliers, and missing values. Twenty-four patients lacking the required preoperative or postoperative laboratory data were excluded during cohort construction. The remaining 650 patients had complete data for the variables included in the reported analyses; therefore, no statistical imputation was required.

The distribution of continuous data was examined visually and with the Shapiro–Wilk test. Variables with an approximately normal distribution are reported as mean ± standard deviation, whereas non-normally distributed data are summarized as median [interquartile range]. Categorical data are presented as *n* (%). Continuous variables were compared with the independent-samples *t*-test or Mann–Whitney U test, and categorical variables with the chi-square or Fisher’s exact test, as appropriate. Statistical significance was defined as a two-sided *p* value < 0.05.

Multiplicity across the six preoperative and postoperative biomarker comparisons was addressed with the Benjamini–Hochberg FDR correction, and adjusted *p* values below 0.05 were regarded as statistically significant.

Associations between hematologic immune-inflammatory indices and the outcomes were assessed using univariable and multivariable logistic regression. Biomarker effects were expressed as odds ratios (ORs) per 1-standard deviation increase with 95% confidence intervals (CIs). Adjustment variables were selected a priori on the basis of clinical relevance, representation of distinct risk domains, and the number of outcome events, rather than by univariable statistical significance or automated stepwise selection. Because only 52 in-hospital deaths occurred, the mortality model was intentionally parsimonious to limit overfitting and was adjusted for STS score, EF, and CPB time. STS score was selected as an integrated measure of baseline operative risk, EF as a direct measure of ventricular functional reserve, and CPB time as an objective measure of intraoperative surgical burden. Age was not entered separately into the mortality model because it is already incorporated into the STS risk estimate, whereas SYNTAX score was omitted to preserve model parsimony in view of the limited number of mortality events. Firth penalized logistic regression was used for mortality analyses to further reduce small-sample bias and potential separation. In contrast, the composite postoperative adverse event occurred in 221 patients, permitting a broader adjustment model. Because STS was developed primarily for operative risk estimation rather than specifically for the heterogeneous composite endpoint evaluated in this study, age and SYNTAX score were additionally included to capture demographic risk and coronary anatomical complexity, respectively. The composite endpoint model therefore included age, EF, STS score, SYNTAX score, and CPB time and was estimated using conventional logistic regression.

Multicollinearity among adjustment covariates was assessed using variance inflation factors (VIFs), with VIF > 5 prespecified as indicating potentially problematic multicollinearity. VIFs were calculated separately for the covariate set used in each outcome model and were also re-evaluated after addition of the postoperative biomarkers.

Discriminatory performance was evaluated using receiver operating characteristic (ROC) analysis. Areas under the curve (AUCs) were reported with DeLong 95% CIs, and optimal cut-offs were determined using the Youden index. Sensitivity, specificity, positive predictive value, negative predictive value, and accuracy were calculated at the optimal threshold. For prediction models, the cut-off corresponded to the predicted probability threshold. Calibration was assessed using the Brier score, calibration intercept, calibration slope, and Hosmer–Lemeshow goodness-of-fit test.

Incremental predictive value was evaluated by adding postoperative biomarkers to prespecified clinical models. For mortality, the clinical model included STS score, EF, and CPB time, with postoperative MLR, PIV, DNI, and the PIV + DNI combination added separately. For the composite endpoint, the clinical model included age, EF, STS score, SYNTAX score, and CPB time, with postoperative PIV, DNI, and their combination evaluated as additional predictors. AUC differences were compared using DeLong’s test. Model fit and prediction error were assessed using the Akaike information criterion (AIC), Bayesian information criterion (BIC), and Brier score. Continuous net reclassification improvement (NRI) and integrated discrimination improvement (IDI) were calculated for prespecified mortality-model comparisons involving postoperative PIV, DNI, and their combination.

Clinical utility was examined using decision curve analysis by comparing the net benefit of the clinical and biomarker-enhanced models across relevant threshold probabilities. An exploratory postoperative Immune-Inflammatory Score was also constructed using the upper-tertile thresholds of postoperative MLR, PIV, and DNI. One point was assigned for each elevated biomarker, resulting in a score ranging from 0 to 3, and observed mortality and composite adverse event rates were compared across score categories.

Given the retrospective observational design, analyses were interpreted as associations rather than causal effects. Propensity score matching was not performed because the study evaluated biomarkers and outcomes within a single surgical cohort rather than the effect of a treatment or intervention.

**Software:**

All statistical analyses were performed using Python (version 3.12; Python Software Foundation, Wilmington, DE, USA). Data processing and cleaning were conducted using pandas and NumPy, statistical analyses were performed using SciPy and statsmodels, and ROC analysis and classification performance assessments were carried out using scikit-learn. DNI was obtained directly from an ADVIA 2120i automated hematology analyzer (Siemens Healthineers, Erlangen, Germany). 

## 3. Results

**Figure 2:**
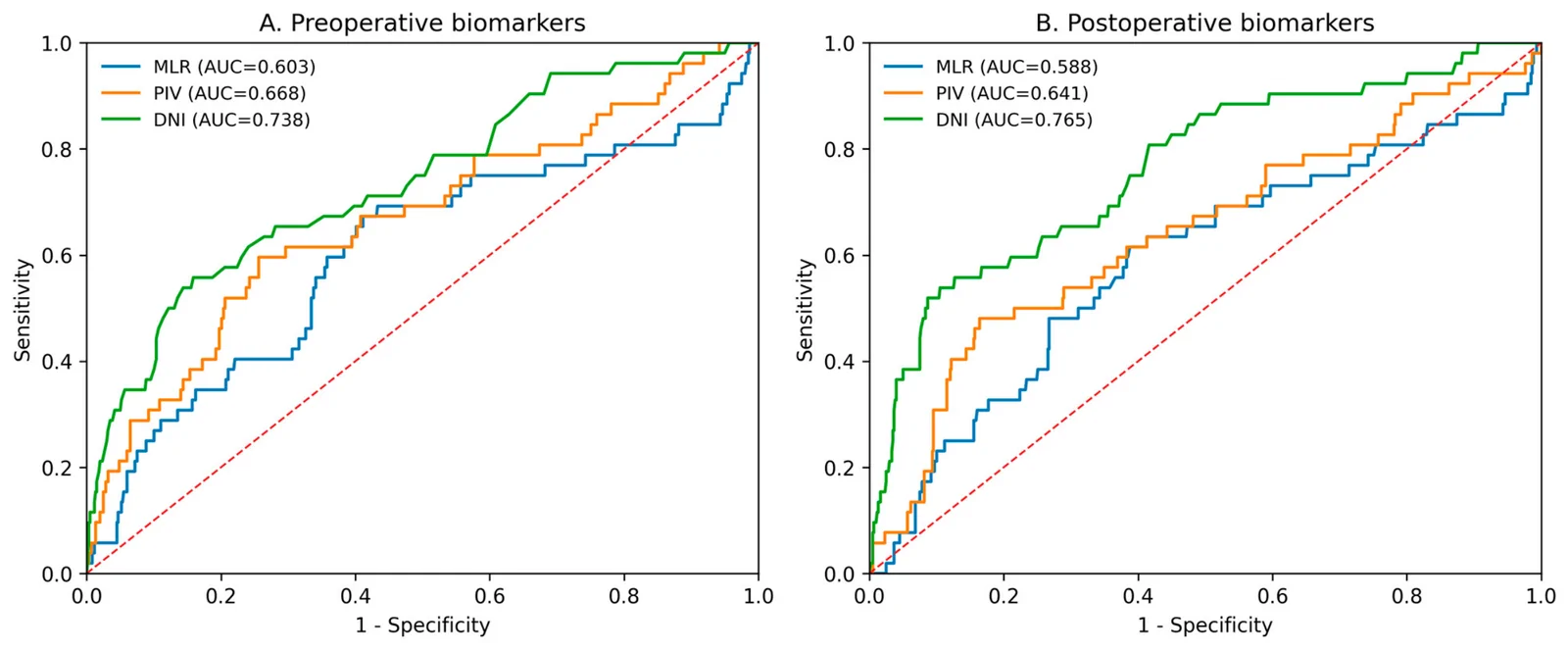
Receiver operating characteristic curves of preoperative and postoperative hematologic immune-inflammatory biomarkers for predicting in-hospital mortality. Panel (**A**) shows ROC curves for preoperative MLR, PIV, and DNI. Panel (**B**) shows ROC curves for postoperative MLR, PIV, and DNI. The dashed diagonal line represents the reference line. MLR = monocyte-to-lymphocyte ratio; PIV = pan-immune-inflammation value; DNI = delta neutrophil index; AUC = area under the receiver operating characteristic curve.

**Figure 3:**
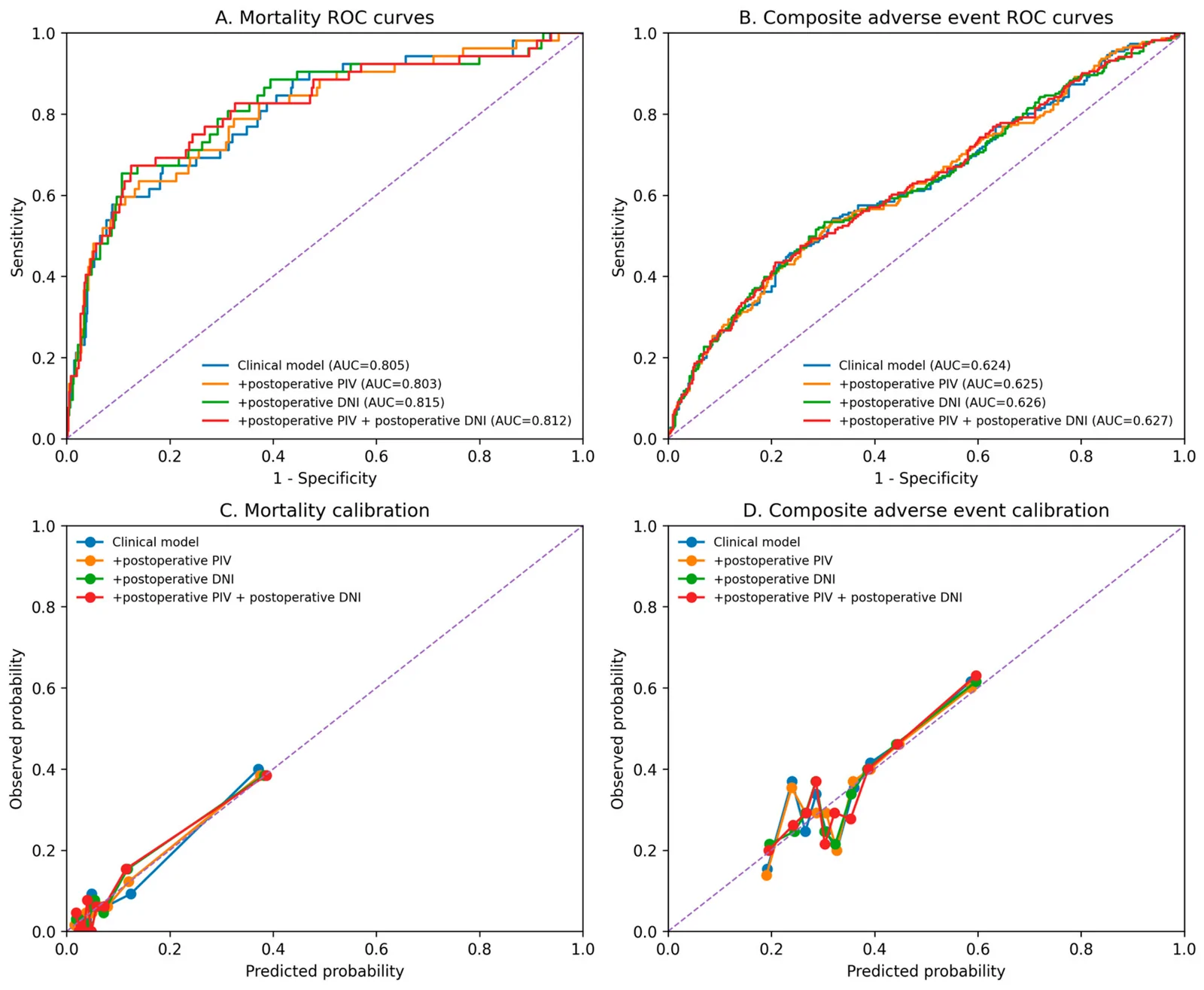
ROC curves and calibration plots for mortality and composite postoperative adverse events. Panel (**A**) shows ROC curves comparing the clinical model and biomarker-enhanced models for predicting in-hospital mortality. Panel (**B**) shows ROC curves comparing the same model set for predicting composite postoperative adverse events. Panel (**C**) shows calibration plots for in-hospital mortality prediction models. Panel (**D**) shows calibration plots for composite postoperative adverse event prediction models. The dashed diagonal line in ROC plots represents the reference line, whereas the dashed diagonal line in calibration plots represents ideal calibration. The mortality clinical model included STS score, EF, and CPB time. The composite postoperative adverse event clinical model included age, EF, STS score, SYNTAX score, and CPB time. PIV = pan-immune-inflammation value; DNI = delta neutrophil index; AUC = area under the receiver operating characteristic curve; STS = Society of Thoracic Surgeons; EF = ejection fraction; CPB = cardiopulmonary bypass.

**Figure 4:**
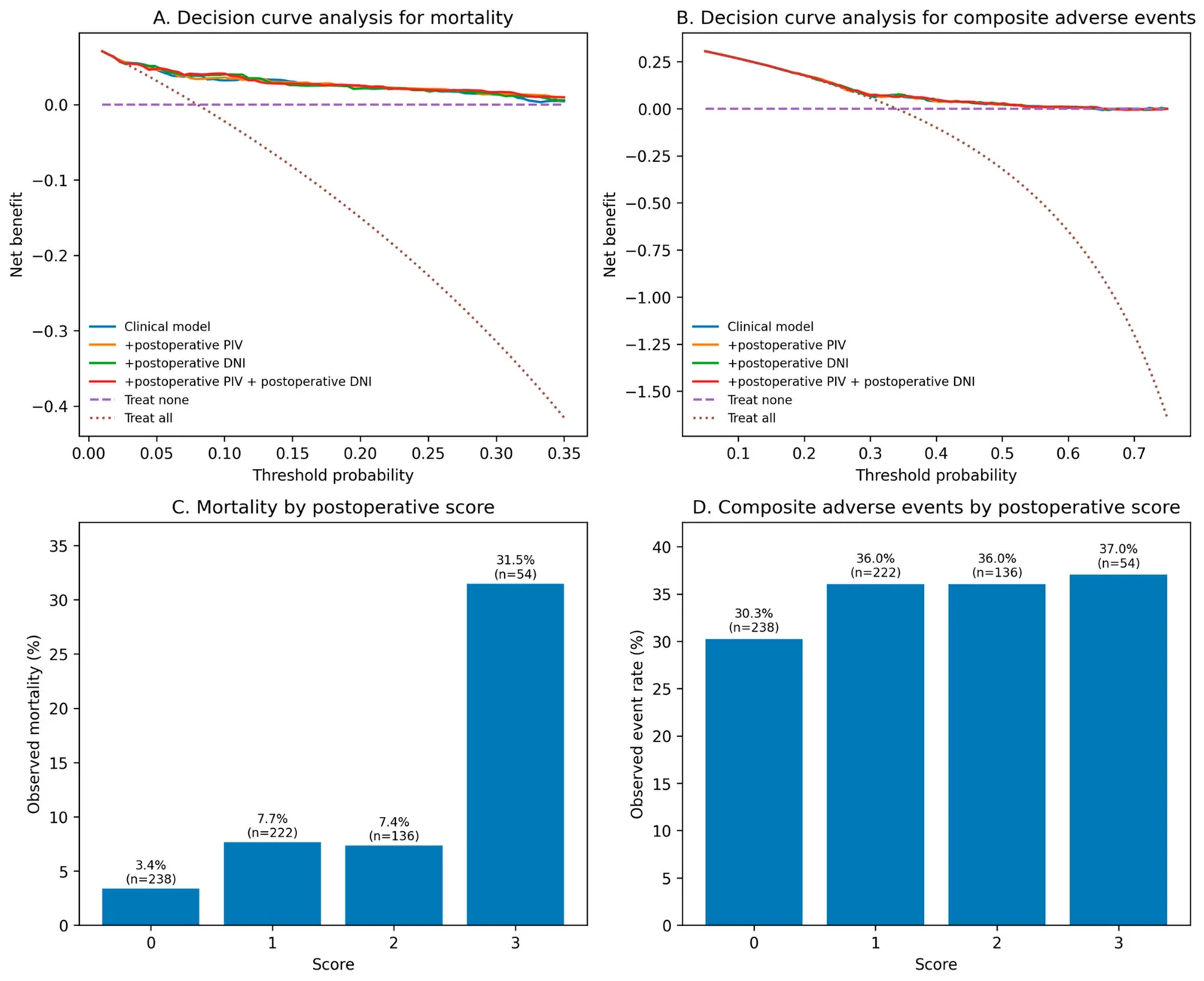
Clinical utility and risk stratification of postoperative immune-inflammatory biomarkers. Panel (**A**) shows decision curve analysis for in-hospital mortality using the clinical model and biomarker-enhanced models. Panel (**B**) shows decision curve analysis for composite postoperative adverse events using the same model comparisons. Panel (**C**) shows observed in-hospital mortality rates across postoperative Immune-Inflammatory Score groups. Panel (**D**) shows observed composite postoperative adverse event rates across postoperative Immune-Inflammatory Score groups. The postoperative Immune-Inflammatory Score was constructed using upper-tertile thresholds for postoperative MLR, PIV, and DNI, with 1 point assigned for each elevated biomarker, yielding a score range from 0 to 3. PIV = pan-immune-inflammation value; DNI = delta neutrophil index; MLR = monocyte-to-lymphocyte ratio.

The final analytic cohort included 650 patients, of whom 598 survived and 52 experienced in-hospital mortality. Composite postoperative adverse events occurred in 221 patients (34.0%). The principal clinical, laboratory, biomarker, regression, and predictive-performance findings are presented in Table 1, Table 2, Table 3, Table 4, Table 5, Table 6, Table 7 and Table 8 and Figure 2, Figure 3 and Figure 4.

Non-survivors had a less favorable baseline clinical and operative risk profile, including older age, lower EF, higher surgical and anatomical risk scores, and longer CPB and aortic cross-clamp times (Table 1).

Both preoperative and postoperative laboratory profiles differed substantially between survivors and non-survivors, particularly with respect to renal function, inflammatory parameters, nutritional status, myocardial injury markers, and hematologic variables (Table 2).

Preoperative and postoperative MLR, PIV, and DNI were significantly higher in non-survivors, and all six comparisons remained significant after FDR correction (Table 3).

Postoperative adverse events and greater cumulative complication burden were significantly more frequent among non-survivors (Table 4).

No problematic multicollinearity was identified among the prespecified adjustment covariates. In the mortality model, VIF values were 2.02 for STS score, 1.19 for EF, and 1.92 for CPB time. In the composite postoperative adverse event model, VIF values were 1.30 for age, 1.32 for EF, 2.73 for STS score, 1.98 for SYNTAX score, and 2.21 for CPB time. All values were well below the prespecified threshold of 5. Multicollinearity remained acceptable after addition of the postoperative biomarkers; even in the model simultaneously containing postoperative PIV and DNI, the maximum VIF was 2.20 for the mortality model and 2.95 for the composite endpoint model. Several immune-inflammatory indices were associated with mortality or composite postoperative adverse events in univariable analyses; however, none remained independently associated with either outcome after multivariable adjustment (Table 5).

Among the individual biomarkers, postoperative DNI showed the highest discrimination for in-hospital mortality. The clinical model showed better overall discrimination, and biomarker-enhanced models produced only small numerical changes in performance (Table 6).

DNI showed the strongest discrimination among both the preoperative and postoperative immune-inflammatory indices (Figure 2).

Addition of postoperative biomarkers did not significantly improve discrimination over the clinical mortality model, and model fit, prediction error, and reclassification measures likewise showed no consistent incremental benefit (Table 7).

For composite postoperative adverse events, addition of postoperative PIV, DNI, or their combination provided negligible incremental discrimination over the clinical model (Table 8).

ROC and calibration analyses showed substantial overlap between the clinical and biomarker-enhanced models, without evidence of a meaningful improvement in discrimination or calibration (Figure 3).

Decision curve analysis showed no consistent net-benefit advantage of the biomarker-enhanced models. Exploratory risk stratification showed the clearest concentration of mortality in patients with the highest postoperative Immune-Inflammatory Score, whereas the gradient for the composite endpoint was modest (Figure 4).

## 4. Discussion

In this single-center retrospective cohort study, we comprehensively evaluated the associations of preoperative and postoperative hematologic immune-inflammatory indices with in-hospital mortality and composite postoperative adverse events in patients undergoing isolated coronary artery bypass grafting. The main finding was that MLR, PIV, and particularly DNI showed significant prognostic signals in unadjusted analyses, with DNI demonstrating the strongest individual discriminatory performance, especially in the postoperative period. Preoperative MLR, PIV, and DNI were higher in non-survivors, and postoperative values of all three indices were also significantly elevated. However, after adjustment for established clinical and operative risk factors, none of the immune-inflammatory indices remained independently associated with mortality or composite postoperative adverse events. These findings indicate that the observed associations between the immune-inflammatory indices and adverse outcomes were largely attenuated after accounting for established clinical and operative risk factors, suggesting limited independent prognostic information beyond the patient’s underlying risk profile and operative burden.

CABG surgery is a complex biological process that physiologically requires a controlled inflammatory response but may transform into maladaptive systemic inflammation in some patients. Surgical trauma, cardiopulmonary bypass, blood contact with extracorporeal surfaces, ischemia–reperfusion injury, endothelial activation, complement and coagulation pathways, and neutrophil activation are among the main determinants of the postoperative inflammatory response [3,4]. In patients with a balanced response, inflammation remains part of tissue repair and hemodynamic adaptation, whereas an excessive or uncontrolled response may contribute to vasoplegia, capillary leakage, pulmonary dysfunction, renal injury, myocardial depression, infectious complications, and multiple organ dysfunction [1,3,4]. Therefore, postoperative immune activation may provide additional biological information when interpreted together with the preoperative risk profile.

In this context, MLR, PIV, and DNI represent different biological aspects of the inflammatory response. MLR reflects the balance between monocyte-mediated innate immune activation and lymphocyte-mediated immune regulation. Increased monocyte counts are associated with atherosclerotic inflammation, tissue injury, and cytokine responses, whereas lymphopenia may reflect surgical stress, cortisol response, and immune dysregulation [8,13]. PIV combines neutrophil, platelet, monocyte, and lymphocyte components and aims to integrate inflammation, platelet activation, innate immune response, and immune suppression within a single index [9,14]. DNI, in contrast, reflects the circulating immature granulocyte fraction and acute neutrophilic bone marrow response [10,15]. This biological distinction may help explain why DNI showed the strongest individual association and discrimination in our cohort.

In our study, preoperative DNI was associated with both mortality and composite postoperative adverse events in univariable analyses, suggesting that patients with a pre-existing activated neutrophilic-inflammatory background may be more vulnerable to surgical stress. Such a profile may reflect subclinical inflammation, advanced atherosclerotic burden, metabolic impairment, reduced renal reserve, or broader biological vulnerability. Postoperative DNI demonstrated an even stronger discriminatory signal for mortality, supporting the concept that the inflammatory response developing after surgery reflects the interaction between baseline vulnerability and operative stress. However, the loss of statistical significance after adjustment indicates that DNI should not be interpreted as an independent determinant of outcome, but rather as a marker that captures part of the same biological and clinical risk continuum represented by established perioperative variables.

The clinical relevance of this relationship is also evident for postoperative adverse events. The secondary outcome consisted of prolonged mechanical ventilation, prolonged intensive care unit stay, prolonged inotropic support, pneumonia, and sepsis. Although these complications are clinically distinct, they converge along a common inflammation–hemodynamics–organ dysfunction axis. Prolonged mechanical ventilation may be associated with pulmonary inflammation, fluid overload, muscle weakness, infection, or hemodynamic instability [16]. Prolonged inotropic support may reflect myocardial depression, vasoplegia, or low cardiac output syndrome [17], whereas pneumonia and sepsis represent more severe disturbances in the balance between immune activation and immune dysfunction [18,19]. In our cohort, these complications were substantially more frequent among non-survivors, supporting their close relationship with early mortality, although the inflammatory indices themselves did not retain independent associations after clinical adjustment.

The prognostic role of hematologic and inflammation-based markers after CABG has previously been investigated using the neutrophil-to-lymphocyte ratio, platelet-to-lymphocyte ratio (PLR), systemic immune-inflammation index, C-reactive protein-to-albumin ratio, and other related indices [20,21,22]. More recently, additional readily available indices have also been explored. Tuncay et al. evaluated the glucose-to-lymphocyte ratio as a prognostic marker in relation to saphenous vein graft stenosis after CABG [23], highlighting the potential relevance of combined metabolic and inflammatory information. In addition, a recent meta-analysis by Li et al. specifically evaluated the association between PLR and prognosis in patients undergoing CABG [24], further supporting continued interest in simpler hematologic indices for postoperative risk assessment. These findings illustrate that a broad range of inexpensive blood-derived indices may reflect overlapping aspects of systemic inflammation and clinical vulnerability after CABG. However, many previous studies have focused on a single biomarker, a single measurement time point, or a specific postoperative outcome. In this context, our findings are important because the more complex immune-inflammatory indices evaluated in the present study did not provide substantial incremental prognostic improvement beyond established clinical and operative risk factors. Thus, increasing biomarker complexity does not necessarily translate into greater predictive value. Future studies directly comparing newer composite indices with simpler established markers such as PLR, NLR, and glucose-to-lymphocyte ratio within the same population may help determine whether any hematologic index provides clinically meaningful incremental information.

Previous studies on MLR have shown that monocyte activation and lymphopenia may be associated with poor prognosis in cardiovascular diseases [8,25]. In coronary artery disease, monocytes are key cellular components of atherosclerotic plaque inflammation and contribute to cytokine production, matrix degradation, and plaque instability through differentiation into tissue macrophages [26]. Low lymphocyte counts may also reflect stress responses and impaired immune regulation. Therefore, the prognostic relevance of MLR in CABG patients is biologically plausible. In our study, however, MLR demonstrated relatively weak discrimination and did not remain significant after multivariable adjustment, suggesting that the monocyte–lymphocyte axis alone may be insufficient to characterize the complex inflammatory response associated with adverse postoperative outcomes.

PIV theoretically represents a broader immune-inflammatory domain. By combining increases in neutrophils, platelets, and monocytes with decreased lymphocytes, PIV has become an attractive marker in conditions where inflammation and thromboinflammation coexist [9,14]. Oncological and cardiovascular studies have reported associations between elevated PIV and adverse prognosis [9,14,27]. In our cohort, both preoperative and postoperative PIV were higher among non-survivors, and PIV showed significant univariable associations with mortality. Nevertheless, these associations were attenuated after adjustment for established clinical variables. This suggests that PIV reflects overall inflammatory burden but provides limited independent information beyond conventional perioperative risk factors. The influence of hemodilution, bleeding, transfusion, cardiopulmonary bypass, and platelet consumption may also affect the stability of postoperative PIV.

Our findings regarding DNI are consistent with previous observations in sepsis, critical illness, and inflammatory states after cardiac surgery [10,25]. Because DNI reflects the release of immature granulocytes into the circulation, it has been associated with bacterial infection, sepsis, severe systemic inflammation, and bone marrow stress [10]. Cardiopulmonary bypass and ischemia–reperfusion injury provide strong stimuli for neutrophil activation and granulocyte release [3,4]. In our study, postoperative DNI showed the highest discrimination among the individual biomarkers for in-hospital mortality, with an AUC of 0.765, compared with 0.738 for preoperative DNI and lower AUCs for MLR and PIV. Among the evaluated indices, DNI showed the strongest individual discriminatory performance; however, its moderate discrimination and lack of an independent association after multivariable adjustment limit its prognostic relevance beyond established clinical and operative risk factors.

One of the important contributions of our study is that biomarkers were evaluated not only according to statistical significance but also according to model performance and clinical usefulness. A difference in biomarker levels between outcome groups does not necessarily imply clinically meaningful predictive improvement. Therefore, ROC analysis, Brier score, calibration metrics, Hosmer–Lemeshow testing, AIC, BIC, DeLong testing, NRI, IDI, and decision curve analysis were used to assess whether biomarker addition meaningfully improved prediction beyond established clinical variables.

The observed in-hospital mortality of 8.0% deserves consideration in the context of the clinical profile of the study population. Although emergency or urgent procedures, redo CABG, off-pump surgery, and concomitant cardiac operations were excluded, elective surgical status should not be equated with low baseline risk. Patients who died were older and had significantly higher STS, EuroSCORE, and SYNTAX scores, lower left ventricular ejection fraction, markedly impaired renal function, lower albumin, and a greater inflammatory burden. They also experienced substantially longer cardiopulmonary bypass and aortic cross-clamp times, indicating an important contribution of intraoperative complexity to the observed outcome. Consistent with these findings, the STS score remained strongly associated with mortality and showed good individual discrimination in our cohort. Nevertheless, the absolute observed mortality was higher than would be suggested by the overall STS risk distribution. This difference should not be interpreted as failure of the STS model to identify higher-risk patients; rather, it highlights the distinction between baseline preoperative risk estimation and the total perioperative risk ultimately encountered by an individual patient. The STS model provides a population-derived preoperative estimate and cannot incorporate subsequent intraoperative factors such as prolonged cardiopulmonary bypass, ischemic time, or the magnitude of the postoperative inflammatory and organ dysfunction response [28]. In addition, differences in case mix, institutional practice, geographic population characteristics, and model calibration may contribute to differences between predicted and observed event rates [29]. Accordingly, STS risk remained clinically informative for risk stratification in the present cohort, but it did not fully account for the excess risk that emerged during the perioperative course. Importantly, the 8.0% mortality observed in the present analytic cohort should not be interpreted as the overall institutional mortality rate for isolated CABG, because this study represents a selected biomarker-analysis cohort rather than an unselected institutional quality registry. The observed rate is higher than contemporary large-scale benchmarks; recent STS data report an in-hospital mortality of approximately 1.7% for isolated CABG, while a large Turkish registry previously reported an in-hospital mortality of 1.23% [30,31]. Conversely, a selected higher-risk Turkish isolated CABG cohort reported an observed mortality of 7.9% [29], illustrating the substantial influence of case mix on crude mortality rates. Therefore, the mortality observed in our cohort should be interpreted in the context of its clinical risk profile, perioperative complexity, and single-center case mix rather than as a benchmark for the institution as a whole.

The baseline mortality model based on STS score, ejection fraction, and cardiopulmonary bypass duration achieved an AUC of 0.805. The greatest numerical gain was observed after inclusion of postoperative DNI, increasing the AUC to 0.815, while simultaneous addition of PIV and DNI yielded an AUC of 0.812. Neither change reached statistical significance, and the accompanying changes in AIC, BIC, Brier score, NRI, and IDI did not show a consistent improvement. Thus, although postoperative DNI contains prognostic information, our findings do not demonstrate a substantial incremental predictive benefit beyond the prespecified clinical model. Rather, the results suggest that the postoperative inflammatory response largely complements, and partly overlaps with, conventional measures of patient and operative risk.

The results for composite postoperative adverse events were even more conservative. The clinical model showed modest discrimination, with an AUC of 0.624, and adding postoperative PIV, DNI, or both resulted in only minimal increases to 0.625, 0.626, and 0.627, respectively. Therefore, the inflammatory biomarkers did not materially improve prediction of the composite endpoint. From a clinical perspective, these findings argue against using DNI or PIV in isolation to trigger interventions. Instead, abnormal values may serve as supportive warning signals that should be interpreted together with hemodynamic status, respiratory course, infection parameters, renal function, and conventional risk factors.

Another aspect of the study was the evaluation of a simple postoperative immune-inflammatory score. The score was constructed using upper-tertile thresholds for postoperative MLR, PIV, and DNI. Mortality rates were 3.4%, 7.7%, 7.4%, and 31.5% across scores of 0, 1, 2, and 3, respectively, demonstrating a marked concentration of mortality in patients with elevation of all three biomarkers. In contrast, composite adverse event rates showed only a modest gradient across score groups. These findings suggest that cumulative inflammatory activation may identify a subgroup with particularly high mortality risk, although the non-linear pattern and exploratory nature of this score preclude its current use as a definitive bedside risk tool.

An important strength of the present analysis is the procedural uniformity of the cohort, which was restricted to isolated CABG. Excluding concomitant valve, aortic, and other major cardiac procedures minimized variation in inflammatory responses attributable to differences in operative type. Second, inflammation was evaluated dynamically using both preoperative and postoperative measurements. Third, MLR, PIV, and DNI were compared within the same population, allowing assessment of their relative prognostic value. Fourth, both a robust primary endpoint of in-hospital mortality and clinically relevant postoperative complications were evaluated. Finally, the statistical approach extended beyond conventional *p* values and included FDR correction, Firth penalized regression, ROC analysis, calibration, model fit, reclassification, and decision curve analysis.

Nevertheless, the findings should be interpreted cautiously. The retrospective, single-center design limits generalizability and does not permit causal inference. Although the cohort included 650 patients, only 52 in-hospital deaths occurred, limiting the complexity and stability of mortality models and justifying the use of Firth penalized regression. Furthermore, biomarker thresholds and the exploratory immune-inflammatory score were derived from the same cohort and therefore require external validation. Postoperative measurements were obtained at a single predefined time point, and serial changes beyond the first 24 h were not evaluated.

From a clinical perspective, the present findings do not support the use of MLR, PIV, or DNI as independent risk-stratification tools after CABG. Although these indices are inexpensive and readily available and may reflect the magnitude of the perioperative inflammatory response, their prognostic information substantially overlapped with established clinical and operative risk factors. Postoperative DNI showed the strongest individual signal, but its incremental contribution to the clinical model was small and statistically non-significant. Therefore, these biomarkers should primarily be regarded as indicators of perioperative inflammatory burden rather than substitutes for established risk assessment.

### Limitations of the Study

This study has several limitations. First, its single-center, retrospective, observational design limits external generalizability and does not permit causal inference. Perioperative management, surgical techniques, intensive care practices, and laboratory measurements reflect the routine practice of a single institution. Nevertheless, restricting the cohort to isolated CABG reduced procedural heterogeneity and improved internal consistency.

Second, all variables were obtained retrospectively from routine clinical records. Although data were checked for consistency, biological plausibility, and missingness, some variability in sampling time, transfusion exposure, fluid balance, hemodilution, and postoperative treatment could not be completely controlled. These factors may particularly influence hemogram-derived indices such as PIV, which incorporates several circulating cell populations.

Third, inflammatory assessment was limited to routinely available hematologic indices. More specific inflammatory biomarkers, including cytokines, procalcitonin, complement activation markers, endothelial injury markers, and cellular immune phenotyping, were not evaluated. Accordingly, the present study was designed to assess practical prognostic biomarkers rather than to characterize the full biological mechanisms underlying postoperative inflammation.

Fourth, although the cohort included 650 patients, only 52 in-hospital deaths occurred. This limited the number of covariates that could be incorporated into mortality models and may still introduce estimation uncertainty. To reduce small-sample bias and model instability, Firth penalized logistic regression and a limited number of clinically prespecified covariates were used. Importantly, none of the evaluated immune-inflammatory indices remained independently associated with mortality or composite postoperative adverse events after adjustment, emphasizing that their prognostic signals should be interpreted as complementary rather than independent.

Fifth, the composite postoperative adverse event included prolonged mechanical ventilation, prolonged intensive care unit stay, prolonged inotropic support, pneumonia, and sepsis. These events differ in clinical severity and pathophysiology, and their individual contributions to the composite outcome may therefore vary. Furthermore, only in-hospital mortality was assessed; 30-day, 90-day, and long-term outcomes were unavailable with sufficient completeness.

Finally, although discrimination, calibration, model fit, reclassification, and decision curve analyses were performed, no external validation cohort was available. Biomarker thresholds and the exploratory postoperative immune-inflammatory score were derived from the same population and may vary according to patient characteristics, laboratory platforms, and institutional practices. In addition, biomarker-enhanced models provided only limited incremental predictive improvement over the prespecified clinical models, further supporting the need for prospective multicenter validation before clinical implementation.

Despite these limitations, the study provides a comprehensive comparison of preoperative and postoperative MLR, PIV, and DNI within a homogeneous isolated CABG cohort. The findings support these readily available hematologic indices, particularly DNI, as potential complementary markers of perioperative risk, while prospective multicenter studies with external validation are required before their routine use in clinical risk prediction.

## 5. Conclusions

In conclusion, preoperative and postoperative MLR, PIV, and DNI were associated with adverse outcomes in unadjusted analyses after isolated CABG, with postoperative DNI showing the strongest individual discrimination for in-hospital mortality. However, none of these indices remained independently associated with mortality or composite postoperative adverse events after adjustment for established clinical and operative risk factors. Furthermore, the addition of postoperative DNI to the clinical mortality model produced only a small and statistically non-significant improvement in discrimination. Therefore, the present findings indicate that these routinely available immune-inflammatory indices have limited incremental prognostic value beyond conventional risk assessment. Their clinical role, if any, should be considered supportive and hypothesis-generating, and further validation is required before they can be incorporated into routine postoperative risk stratification.

## Figures and Tables

**Table 1:** Baseline, Risk Score, and Operative Characteristics According to Mortality (*n* = 650).

Variable	Overall Cohort (*n* = 650)	Survivors (*n* = 598)	Non-Survivors (*n* = 52)	*p* Value	SMD
**Clinical characteristics**					
Age, years	60.00 [55.00–65.00]	60.00 [54.00–64.00]	67.00 [62.00–70.00]	**<0.001**	0.885
Male sex, *n* (%)	471 (72.5)	437 (73.1)	34 (65.4)	0.234	0.167
EF, %	55.00 [50.00–56.75]	55.00 [50.00–57.00]	50.00 [45.00–55.00]	**0.002**	0.551
Diabetes mellitus, *n* (%)	513 (78.9)	470 (78.6)	43 (82.7)	0.487	0.104
Hypertension, *n* (%)	352 (54.2)	314 (52.5)	38 (73.1)	**0.004**	0.436
**Risk scores**					
STS score	2.26 [1.42–3.01]	2.15 [1.38–2.92]	3.79 [2.57–5.15]	**<0.001**	1.375
EuroSCORE	2.00 [1.00–3.00]	2.00 [1.00–3.00]	4.00 [2.75–7.00]	**<0.001**	1.345
SYNTAX score	24.1 ± 7.1	23.5 ± 6.8	30.1 ± 8.4	**<0.001**	0.957
**Preoperative laboratory variables**					
HbA1c, %	7.10 [5.90–7.80]	7.10 [5.90–7.80]	7.00 [6.30–7.58]	0.917	0.011
LDL, mg/dL	113.00 [91.00–138.00]	113.00 [91.00–138.00]	114.00 [91.50–138.25]	0.851	0.058
Total cholesterol, mg/dL	198.2 ± 38.7	198.6 ± 38.8	193.7 ± 37.5	0.383	0.126
HDL, mg/dL	40.00 [34.00–46.00]	40.00 [34.00–46.00]	38.00 [31.00–45.00]	0.157	0.188
**Operative characteristics**					
CPB time, min	110.00 [98.00–122.00]	109.00 [98.00–120.00]	142.50 [113.75–159.25]	**<0.001**	1.345
Cross-clamp time, min	68.00 [60.00–76.00]	67.50 [59.00–75.75]	87.00 [70.00–100.25]	**<0.001**	1.241

Continuous variables are presented as mean ± standard deviation or median [interquartile range], according to distributional characteristics, and categorical variables are presented as number and percentage. Statistical tests applied include the independent-samples Student’s *t*-test or Mann–Whitney U test for continuous variables, according to distributional characteristics, and the Chi-square test or Fisher’s exact test for categorical variables, as appropriate. In the table, statistically significant *p* values are marked in bold. Values less than 0.05 were considered statistically significant. SMD indicates the standardized mean difference between survivors and non-survivors. EF = ejection fraction; HT = hypertension; STS = Society of Thoracic Surgeons; EuroSCORE = European System for Cardiac Operative Risk Evaluation; SYNTAX = Synergy Between Percutaneous Coronary Intervention With Taxus and Cardiac Surgery; HbA1c = glycated hemoglobin; LDL = low-density lipoprotein; HDL = high-density lipoprotein; GFR = glomerular filtration rate; CRP = C-reactive protein; CPB = cardiopulmonary bypass; SMD = standardized mean difference.

**Table 2:** Preoperative and Postoperative Laboratory Profile According to Mortality (*n* = 650).

Variable	Overall Cohort (*n* = 650)	Survivors (*n* = 598)	Non-Survivors (*n* = 52)	*p* Value	SMD
**Preoperative laboratory variables**					
Urea	37.30 [27.47–45.90]	35.80 [26.38–44.80]	52.30 [45.33–67.60]	**<0.001**	1.329
Creatinine	1.10 [0.89–1.33]	1.07 [0.87–1.29]	1.45 [1.21–2.25]	**<0.001**	1.476
GFR	72.95 [54.60–95.50]	74.95 [57.65–96.20]	42.40 [27.07–62.75]	**<0.001**	1.070
Sodium	139.15 ± 2.39	139.21 ± 2.35	138.51 ± 2.71	**0.042**	0.295
Potassium	4.27 ± 0.36	4.28 ± 0.36	4.25 ± 0.38	0.569	0.082
ALT	25.20 [20.70–29.00]	25.10 [20.60–28.65]	26.60 [22.30–30.20]	**0.032**	0.427
AST	28.85 [24.80–34.00]	28.70 [24.70–33.90]	31.50 [27.88–37.73]	**0.002**	0.380
CRP	12.30 [7.50–17.80]	11.95 [7.30–16.80]	22.95 [13.25–35.23]	**<0.001**	1.099
Albumin	3.93 [3.60–4.23]	3.96 [3.63–4.27]	3.29 [2.84–3.69]	**<0.001**	1.338
Troponin	0.05 [0.03–0.18]	0.05 [0.03–0.11]	0.10 [0.04–0.93]	**0.002**	0.668
CK-MB	4.60 [2.80–19.22]	4.50 [2.70–13.18]	6.05 [3.65–44.42]	**0.003**	0.592
Hemoglobin	12.84 ± 1.46	12.90 ± 1.44	12.18 ± 1.52	**<0.001**	0.500
Hematocrit	38.82 ± 4.69	38.98 ± 4.64	36.98 ± 4.82	**0.003**	0.430
WBC	9.35 [8.26–10.47]	9.34 [8.22–10.40]	9.60 [8.72–11.32]	0.056	0.360
Platelet	239.65 ± 58.14	238.54 ± 57.43	252.42 ± 65.00	0.099	0.239
PDW	13.50 ± 1.49	13.47 ± 1.46	13.85 ± 1.76	0.074	0.259
RDW	14.12 ± 1.24	14.04 ± 1.21	15.00 ± 1.19	**<0.001**	0.795
DNI	0.51 [0.33–0.72]	0.50 [0.32–0.69]	0.85 [0.51–1.14]	**<0.001**	1.123
MPV	9.75 ± 0.83	9.74 ± 0.82	9.78 ± 0.95	0.722	0.052
Monocyte	0.70 [0.57–0.86]	0.70 [0.58–0.87]	0.70 [0.55–0.82]	0.504	0.090
Neutrophil	5.76 [4.93–6.77]	5.74 [4.88–6.69]	6.64 [5.60–7.62]	**<0.001**	0.751
Lymphocyte	2.41 [1.96–3.00]	2.43 [1.99–3.04]	1.94 [1.48–2.55]	**<0.001**	0.529
**Postoperative laboratory variables**					
Urea	48.25 [36.70–60.45]	47.50 [35.25–58.45]	80.75 [55.00–99.22]	**<0.001**	1.407
Creatinine	1.25 [1.06–1.62]	1.24 [1.03–1.54]	1.92 [1.49–3.00]	**<0.001**	1.626
GFR	61.00 [43.52–80.78]	63.90 [46.60–84.30]	30.25 [19.90–49.30]	**<0.001**	1.078
Sodium	138.10 ± 3.35	138.20 ± 3.35	137.00 ± 3.22	**0.013**	0.360
Potassium	4.41 ± 0.56	4.41 ± 0.56	4.38 ± 0.52	0.654	0.065
ALT	39.00 [30.40–51.68]	38.40 [30.32–50.20]	54.10 [35.38–69.42]	**<0.001**	0.912
AST	51.75 [39.30–68.95]	50.55 [38.92–67.67]	65.85 [53.02–84.08]	**<0.001**	0.656
CRP	93.75 [74.03–124.25]	92.30 [73.30–119.38]	131.35 [91.85–208.07]	**<0.001**	1.147
Albumin	3.43 [3.03–3.77]	3.50 [3.15–3.81]	2.66 [2.25–3.05]	**<0.001**	1.462
Troponin	2.47 [1.62–3.37]	2.42 [1.57–3.22]	3.87 [2.54–5.63]	**<0.001**	1.065
CK-MB	55.50 [36.20–79.20]	53.40 [35.40–73.00]	100.15 [45.65–133.65]	**<0.001**	1.020
Hemoglobin	11.16 ± 1.60	11.22 ± 1.56	10.40 ± 1.79	**<0.001**	0.521
Hematocrit	33.37 ± 4.98	33.57 ± 4.84	31.10 ± 5.99	**<0.001**	0.500
WBC	13.86 [12.00–15.85]	13.71 [11.91–15.65]	17.16 [13.98–21.33]	**<0.001**	1.104
Platelet	180.00 [141.00–226.75]	180.00 [142.00–225.00]	175.50 [122.75–239.25]	0.840	0.024
PDW	13.99 ± 1.65	13.93 ± 1.60	14.72 ± 1.95	**<0.001**	0.486
RDW	14.49 ± 1.28	14.40 ± 1.24	15.45 ± 1.34	**<0.001**	0.832
DNI	1.11 [0.76–1.62]	1.05 [0.74–1.56]	2.33 [1.24–3.06]	**<0.001**	1.296
MPV	9.91 ± 1.01	9.90 ± 1.00	10.07 ± 1.10	0.252	0.166
Monocyte	0.84 [0.65–1.06]	0.84 [0.64–1.05]	0.88 [0.71–1.11]	0.216	0.200
Neutrophil	11.69 [9.96–13.72]	11.57 [9.89–13.34]	15.42 [11.96–18.41]	**<0.001**	1.119
Lymphocyte	0.72 [0.57–1.01]	0.74 [0.58–1.02]	0.64 [0.55–0.95]	0.159	0.068

Continuous variables are presented as mean ± standard deviation or median [interquartile range], according to distributional characteristics. Statistical tests applied include the independent-samples Student’s *t*-test for approximately normally distributed continuous variables and the Mann–Whitney U test for non-normally distributed continuous variables. In the table, statistically significant *p* values are marked in bold. Values less than 0.05 were considered statistically significant. SMD indicates the standardized mean difference between survivors and non-survivors. GFR = glomerular filtration rate; ALT = alanine aminotransferase; AST = aspartate aminotransferase; CRP = C-reactive protein; CK-MB = creatine kinase-myocardial band; WBC = white blood cell count; PDW = platelet distribution width; RDW = red cell distribution width; DNI = delta neutrophil index; MPV = mean platelet volume; SMD = standardized mean difference.

**Table 3:** Hematologic Immune-Inflammatory Indices According to Mortality (*n* = 650).

Variable	Survivors (*n* = 598)	Non-Survivors (*n* = 52)	*p* Value	FDR-Adjusted *p* Value
**Preoperative indices**				
MLR	0.29 [0.23–0.36]	0.33 [0.26–0.47]	**0.014**	**0.017**
PIV	367.87 [263.04–535.93]	573.89 [338.22–870.93]	**<0.001**	**<0.001**
DNI	0.50 [0.32–0.69]	0.85 [0.51–1.14]	**<0.001**	**<0.001**
**Postoperative indices**				
MLR	1.10 [0.85–1.43]	1.28 [0.94–1.69]	**0.035**	**0.035**
PIV	2285.54 [1444.79–3282.28]	3279.47 [1944.41–4637.05]	**<0.001**	**0.001**
DNI	1.05 [0.74–1.56]	2.33 [1.24–3.06]	**<0.001**	**<0.001**

Continuous variables are presented as median [interquartile range]. Statistical comparisons between survivors and non-survivors were performed using the Mann–Whitney U test. False discovery rate adjustment was performed using the Benjamini–Hochberg method across the six hematologic immune-inflammatory comparisons. In the table, statistically significant *p* values and FDR-adjusted *p* values are marked in bold. Values less than 0.05 were considered statistically significant. MLR = monocyte-to-lymphocyte ratio; PIV = pan-immune-inflammation value; DNI = delta neutrophil index; FDR = false discovery rate.

**Table 4:** Postoperative Adverse Events and Complication Burden According to Mortality (*n* = 650).

Variable	Overall Cohort (*n* = 650)	Survivors (*n* = 598)	Non-Survivors (*n* = 52)	*p* Value	OR for Mortality
Prolonged mechanical ventilation	59 (9.1)	46 (7.7)	13 (25.0)	**<0.001**	4.00 (1.99–8.02)
Prolonged ICU stay	77 (11.8)	60 (10.0)	17 (32.7)	**<0.001**	4.36 (2.30–8.24)
Prolonged inotropic support	121 (18.6)	95 (15.9)	26 (50.0)	**<0.001**	5.29 (2.95–9.52)
Pneumonia	52 (8.0)	38 (6.4)	14 (26.9)	**<0.001**	5.43 (2.71–10.88)
Sepsis	55 (8.5)	44 (7.4)	11 (21.2)	**0.002**	3.38 (1.62–7.03)
Composite postoperative adverse event	221 (34.0)	181 (30.3)	40 (76.9)	**<0.001**	7.68 (3.94–14.98)
**Complication burden, *n* (%)**				**<0.001**	
0 adverse events	429 (66.0)	417 (69.7)	12 (23.1)		Reference
1 adverse event	137 (21.1)	118 (19.7)	19 (36.5)	**<0.001**	5.60 (2.64–11.86)
≥2 adverse events	84 (12.9)	63 (10.5)	21 (40.4)	**<0.001**	11.58 (5.43–24.70)

Categorical variables are presented as number and percentage. Statistical comparisons between survivors and non-survivors were performed using the Chi-square test or Fisher’s exact test, as appropriate. Odds ratios for mortality were calculated from 2 × 2 contingency tables, with patients without the corresponding adverse event used as the reference category. For complication burden, patients with 0 adverse events were used as the reference group. In the table, statistically significant *p* values are marked in bold. Values less than 0.05 were considered statistically significant. OR = odds ratio; ICU = intensive care unit.

**Table 5:** Univariable and Multivariable Adjusted Logistic Regression Analyses for In-Hospital Mortality and Composite Postoperative Adverse Events (*n* = 650).

Variable	Univariable OR	95% CI	*p* Value	Adjusted OR	95% CI	*p* Value
**A. Mortality**						
Preoperative MLR	1.31	1.09–1.58	**0.005**	1.15	0.91–1.45	0.229
Postoperative MLR	1.22	0.96–1.55	0.110	1.22	0.95–1.56	0.119
Preoperative PIV	1.58	1.28–1.95	**<0.001**	1.17	0.92–1.49	0.189
Postoperative PIV	1.53	1.22–1.91	**<0.001**	1.20	0.94–1.54	0.147
Preoperative DNI	2.39	1.85–3.09	**<0.001**	1.30	0.92–1.83	0.135
Postoperative DNI	2.37	1.87–3.01	**<0.001**	1.29	0.90–1.85	0.166
**B. Composite postoperative adverse event**						
Preoperative MLR	1.12	0.96–1.31	0.156	1.03	0.87–1.22	0.765
Postoperative MLR	0.96	0.81–1.13	0.585	0.93	0.79–1.11	0.432
Preoperative PIV	1.19	1.01–1.40	**0.033**	1.00	0.83–1.19	0.972
Postoperative PIV	1.06	0.90–1.24	0.484	0.95	0.80–1.12	0.536
Preoperative DNI	1.33	1.13–1.57	**<0.001**	1.01	0.83–1.25	0.891
Postoperative DNI	1.39	1.18–1.64	**<0.001**	1.09	0.88–1.35	0.450

Odds ratios are reported per 1-standard deviation increase in each hematologic immune-inflammatory index. Univariable and multivariable logistic regression analyses were performed for in-hospital mortality and composite postoperative adverse events. For the mortality endpoint (52 events), Firth penalized logistic regression was retained to reduce small-sample bias, and the adjusted model included STS score, EF, and CPB time. For the composite postoperative adverse event endpoint, conventional logistic regression was used, and the adjusted model included age, EF, STS score, SYNTAX score, and CPB time. No evidence of problematic multicollinearity was observed in either adjustment model (all VIFs < 3). In the table, statistically significant *p* values are marked in bold. Values less than 0.05 were considered statistically significant. OR = odds ratio; CI = confidence interval; MLR = monocyte-to-lymphocyte ratio; PIV = pan-immune-inflammation value; DNI = delta neutrophil index; STS = Society of Thoracic Surgeons; EF = ejection fraction; CPB = cardiopulmonary bypass; SYNTAX = Synergy Between Percutaneous Coronary Intervention With Taxus and Cardiac Surgery.

**Table 6:** Discrimination and Calibration Performance of Biomarkers and Prediction Models for In-Hospital Mortality (*n* = 650).

Variable	AUC	95% CI	Optimal Cut-Off	Youden İndex	Sensitivity	Specificity	PPV	NPV	Accuracy	Brier Score	Calibration İntercept	Calibration Slope	Hosmer– Lemeshow *p*
**Biomarkers**													
Preoperative MLR	0.603	0.511–0.695	≥0.306	0.262	0.673	0.589	0.125	0.954	0.595	0.073	−0.012	0.993	0.022
Postoperative MLR	0.588	0.499–0.678	≥1.200	0.227	0.615	0.612	0.121	0.948	0.612	0.073	−0.016	0.950	0.350
Preoperative PIV	0.668	0.583–0.753	≥527.379	0.340	0.596	0.744	0.168	0.955	0.732	0.071	−0.010	1.023	0.190
Postoperative PIV	0.641	0.554–0.729	≥3745.342	0.317	0.481	0.836	0.203	0.949	0.808	0.072	−0.015	0.997	0.028
Preoperative DNI	0.738	0.661–0.814	≥0.780	0.399	0.558	0.841	0.234	0.956	0.818	0.065	−0.013	1.009	0.411
Postoperative DNI	0.765	0.692–0.838	≥2.220	0.433	0.538	0.895	0.308	0.957	0.866	0.065	−0.013	1.008	0.636
**Prediction models**													
Clinical model	0.805	0.737–0.872	≥0.128	0.499	0.596	0.903	0.348	0.963	0.878	0.062	−0.027	1.014	0.585
Clinical model + postoperative MLR	0.803	0.735–0.871	≥0.117	0.489	0.596	0.893	0.326	0.962	0.869	0.061	−0.033	1.015	0.136
Clinical model + postoperative PIV	0.803	0.735–0.872	≥0.102	0.494	0.635	0.860	0.282	0.964	0.842	0.061	−0.032	1.017	0.997
Clinical model + postoperative DNI	0.815	0.747–0.884	≥0.113	0.547	0.654	0.893	0.347	0.967	0.874	0.062	−0.035	1.017	0.415
Clinical model + postoperative PIV + postoperative DNI	0.812	0.742–0.882	≥0.101	0.548	0.673	0.875	0.318	0.969	0.858	0.061	−0.039	1.020	0.091

ROC analysis was performed to assess the discriminatory performance of individual biomarkers and prediction models for in-hospital mortality. AUC values are presented with DeLong 95% confidence intervals. Optimal cut-off values were determined using the Youden index. Sensitivity, specificity, PPV, NPV, and accuracy were calculated at the optimal cut-off. For prediction models, the optimal cut-off represents the predicted probability threshold. Calibration performance was evaluated using the Brier score, calibration intercept, calibration slope, and Hosmer–Lemeshow goodness-of-fit test. The clinical model included STS score, EF, and CPB time. Mortality prediction models were estimated using Firth penalized logistic regression. AUC = area under the receiver operating characteristic curve; CI = confidence interval; PPV = positive predictive value; NPV = negative predictive value; MLR = monocyte-to-lymphocyte ratio; PIV = pan-immune-inflammation value; DNI = delta neutrophil index; STS = Society of Thoracic Surgeons; EF = ejection fraction; CPB = cardiopulmonary bypass.

**Table 7:** Incremental Predictive Value Over Conventional Clinical Risk Model for In-Hospital Mortality (*n* = 650).

Model	AUC	ΔAUC	DeLong *p* Value	AIC	BIC	Brier Score	NRI	IDI
Clinical model	0.805	Reference	Reference	299.42	317.33	0.062	Reference	Reference
Clinical model + postoperative MLR	0.803	−0.0018	0.768	299.39	321.78	0.061	—	—
Clinical model + postoperative PIV	0.803	−0.0014	0.827	299.36	321.75	0.061	0.197	0.009
Clinical model + postoperative DNI	0.815	+0.0105	0.171	299.52	321.90	0.062	0.038	0.005
Clinical model + postoperative PIV + postoperative DNI	0.812	+0.0074	0.439	299.30	326.16	0.061	0.120	0.014

Incremental predictive performance was evaluated by comparing each biomarker-enhanced model with the conventional clinical risk model. The clinical model included STS score, EF, and CPB time. ΔAUC represents the absolute change in AUC compared with the clinical model. DeLong’s test was used to compare correlated AUC values. AIC and BIC were used to assess model fit, with lower values indicating better performance after penalizing model complexity. Brier score was used to quantify overall prediction error. Continuous NRI and IDI were calculated for prespecified comparisons involving postoperative PIV, postoperative DNI, and their combined addition to the clinical model. AUC = area under the receiver operating characteristic curve; ΔAUC = change in AUC; AIC = Akaike information criterion; BIC = Bayesian information criterion; NRI = net reclassification improvement; IDI = integrated discrimination improvement; MLR = monocyte-to-lymphocyte ratio; PIV = pan-immune-inflammation value; DNI = delta neutrophil index; STS = Society of Thoracic Surgeons; EF = ejection fraction; CPB = cardiopulmonary bypass.

**Table 8:** Discrimination and Calibration Performance of Prediction Models for Composite Postoperative Adverse Events (*n* = 650).

Model	AUC	95% CI	Optimal Cut-Off	Youden İndex	Sensitivity	Specificity	PPV	NPV	Accuracy	Brier Score	Calibration İntercept	Calibration Slope	Hosmer– Lemeshow *p*
Clinical model	0.624	0.578–0.670	0.345	0.224	0.543	0.681	0.467	0.743	0.634	0.212	0.000	1.000	0.085
Clinical model + postoperative PIV	0.625	0.579–0.672	0.338	0.219	0.538	0.681	0.465	0.741	0.632	0.212	−0.000	1.000	0.200
Clinical model + postoperative DNI	0.626	0.579–0.672	0.343	0.231	0.534	0.697	0.476	0.744	0.642	0.212	−0.000	1.000	0.495
Clinical model + postoperative PIV + postoperative DNI	0.627	0.581–0.673	0.377	0.227	0.434	0.793	0.519	0.731	0.671	0.212	0.000	1.000	0.507

ROC analysis was performed to evaluate the discriminatory performance of prediction models for composite postoperative adverse events. AUC values are presented with DeLong 95% confidence intervals. Optimal cut-off values were determined using the Youden index and represent the predicted probability threshold for each model. Sensitivity, specificity, PPV, NPV, and accuracy were calculated at the optimal cut-off. Calibration performance was assessed using the Brier score, calibration intercept, calibration slope, and Hosmer–Lemeshow goodness-of-fit test. The clinical model included age, EF, STS score, SYNTAX score, and CPB time. Conventional logistic regression was used for the composite endpoint. AUC = area under the receiver operating characteristic curve; CI = confidence interval; PPV = positive predictive value; NPV = negative predictive value; PIV = pan-immune-inflammation value; DNI = delta neutrophil index; EF = ejection fraction; STS = Society of Thoracic Surgeons; SYNTAX = Synergy Between Percutaneous Coronary Intervention With Taxus and Cardiac Surgery; CPB = cardiopulmonary bypass.

## Data Availability

The data supporting the findings of this study are available from the corresponding author upon reasonable request. The raw dataset is not publicly available because of institutional regulations, ethical restrictions, and the retrospective use of anonymized clinical records. Data may be shared for academic and research purposes subject to appropriate institutional and ethical approval.

## Abbreviations

CABG	coronary artery bypass grafting
MLR	monocyte-to-lymphocyte ratio
PIV	pan-immune-inflammation value
DNI	delta neutrophil index
CPB	cardiopulmonary bypass
ICU	intensive care unit
STS	Society of Thoracic Surgeons
EuroSCORE	European System for Cardiac Operative Risk Evaluation
SYNTAX	Synergy Between Percutaneous Coronary Intervention With Taxus and Cardiac Surgery
EF	ejection fraction
GFR	glomerular filtration rate
CRP	C-reactive protein
ROC	receiver operating characteristic
AUC	area under the receiver operating characteristic curve
OR	odds ratio
CI	confidence interval
PPV	positive predictive value
NPV	negative predictive value
FDR	false discovery rate
AIC	Akaike information criterion
BIC	Bayesian information criterion
NRI	net reclassification improvement
IDI	integrated discrimination improvement
